# Picric Acid Test for Albumen

**Published:** 1883-09

**Authors:** 


THE PICRIC ACID TEST FOR ALBUMEN.—Dr. George Johnson, M.D., F.R.S., Professor of Clinical Medicine, Senior Physician to King's College Hospital, writes:

It should always be borne in mind that, in testing for albumen, the *picric acid must be in excess.* A few drops of a saturated solution of picric acid in a highly albuminous specimen will form a coagulum, which is quickly re-dissolved. When urine contains much albumen, it should be mixed with its own volume of the picric acid solution; and in testing a fresh specimen, it is better to begin by adding an equal volume of the test liquid.

One difference between picric acid and nitric acid as tests for albumen is, that whereas an excess of nitric acid, especially when the urine is heated, will entirely re-dissolve the previously precipitated albumen, no excess of picric acid will re-dissolve the precipitate which it has once found in an albuminous solution. Picric acid solution on the surface of the urine is applicable only for the detection of a minute trace of albumen. For this purpose, in my paper read at the Clinical Society, I advise that a column of urine four inches in height should be poured into a six-inch test-tube, and upon this one inch of the picric acid solution. The result is that the upper layer of the urine is mixed with about its own volume of the test liquid; and if albumen is present, the stained portion of the urine is instantly rendered more or less opalescent, and thus contrasts with the unstained and transparent urine below. If the picric acid solution were allowed to flow so gently on to the surface of the urine as merely to come in contact and not to become mixed with its upper portion, the albumen, if present, would not be detected, or it would be indicated only after an interval of some minutes, when the two liquids had become mixed by slow diffusion. There must be an actual mixture in equal proportions, and not merely contact of the two liquids, to ensure the action of the test.

The slight opalescence caused by the picric acid solution in a sample of urine which contains a mere trace of albumen is always increased by the application of heat. So that, if the flame of a spirit-lamp be applied to the upper part of the opalescent column, this will become more opaque than the lower part, which had not been exposed to heat. I now invariably apply heat to a specimen of urine which has been rendered opaque, or more or less coagulated, by picric acid; my chief reason for this practice is to ascertain if peptones ever appear in the urine. In a paper published in the *Journal* March 31st, p. 614, I have shown that, whereas the albuminous precipitate with picric acid is rendered more opaque and coherent by heat, the precipitate which picric acid gives with peptones is entirely re-dissolved by heat, considerably below the boiling point.

The microscope alone would serve to distinguish the precipitate caused by picric acid with peptones, with urates, and with albumen respectively.

The precipitate recently thrown down with artificially prepared peptone appears under the microscope quite homogeneous, and free from solid particles; but when the precipitate, having been dissolved by heat, re-forms on cooling, it seems to consist of numerous very minute, apparently globular particles, in which the so-called "Brunonian movement" is very active. The microscopic appearances of uric acid and urates are so well known as to need no description.

The precipitate produced by picric acid with albumen presents irregular clusters of granular material, which appear much larger and more opaque after the application of heat. According to my experience, a deposit of uric acid and urates is about as rare a result of adding picric acid to urine as a similar deposit caused by nitric acid; and hitherto I have met with no specimen of urine in which the presence of peptones has been indicated. A deposit thrown down by picric acid and re-dissolve dby heat, may pretty safely be assumed to consist of urates, but in any case of doubt, the addition of Fehling's solution and the microscopic appearances will at once serve to distinguish between precipitated peptones and urates.—*British Medical Journal.*

